# Amygdala Nuclei Volumes Are Selectively Associated With Social Network Size in Homeless and Precariously Housed Persons

**DOI:** 10.3389/fnbeh.2020.00097

**Published:** 2020-06-16

**Authors:** Paul W. Jones, Allen E. Thornton, Andrea A. Jones, Verena M. Knerich, Donna J. Lang, Melissa L. Woodward, William J. Panenka, Wayne Su, Alasdair M. Barr, Tari Buchanan, William G. Honer, Kristina M. Gicas

**Affiliations:** ^1^Department of Psychology, Simon Fraser University, Burnaby, BC, Canada; ^2^Department of Psychiatry, University of British Columbia, Vancouver, BC, Canada; ^3^Department of Computer Science, Ludwig Maximilians University, Munich, Germany; ^4^Department of Radiology, University of British Columbia, Vancouver, BC, Canada; ^5^Department of Anesthesiology, Pharmacology, and Therapeutics, University of British Columbia, Vancouver, BC, Canada; ^6^Department of Psychology, York University, Toronto, ON, Canada

**Keywords:** social network, homelessness, marginalization, amygdala, neuroimaging

## Abstract

**Objective**: The amygdala is a brain region comprised of a group of functionally distinct nuclei that play a central role in social behavior. In homeless and precariously housed individuals, high rates of multimorbidity, and structural aspects of the environment may dysregulate social functioning. This study examined the neurobiological substrates of social connection in homeless and precariously housed persons by examining associations between amygdala nuclei volumes and social network size.

**Methods**: Sixty participants (mean age 43.6 years; 73.3% male) were enrolled from an ongoing study of homeless and precariously housed adults in Vancouver, Canada. Social network size was assessed using the Arizona Social Support Interview Schedule. Amygdala nuclei volumes were extracted from anatomic T1-weighted MRI data. The central and basolateral amygdala nuclei were selected as they are implicated in anxiety-related and social behaviors. The hippocampus was included as a control brain region. Multivariable regression analysis investigated the relationship between amygdala nuclei volumes and social network size.

**Results**: After controlling for age, sex, and total brain volume, individuals with the larger amygdala and central nucleus volumes had a larger network size. This association was not observed for the basolateral amygdala complex, though subsequent analysis found the basal and accessory basal nuclei of the basolateral amygdala were significantly associated with social network size. No association was found for the lateral amygdala nucleus or hippocampus.

**Conclusions**: These findings suggest that select amygdala nuclei may be differentially involved in the social connections of persons with multimorbid illness and social marginalization.

## Introduction

Humans are inherently social beings. Research suggests that social connectedness or the feeling of belonging to a group is associated with better overall health and a decreased burden on the healthcare system (Uchino, 2006), whereas social isolation has been linked with greater cognitive decline and increased risk for Alzheimer’s disease (Wilson et al., 2007). Social network characteristics such as size and network density (interconnectedness among members) have been associated with frequency of hospitalization (Clinton et al., 1998), as well as overall mental and psychological health (Saeri et al., 2018). Social networks may be particularly important in homeless and precariously housed individuals, as the network composition and structure modifies life experiences, and affects access to support from network members, shelters, and other support providers (Green et al., 2013). Unfortunately, homeless and precariously housed persons typically have small and fragmented social networks and are highly susceptible to social isolation and loneliness (Hawkins and Abrams, 2007; Crawley et al., 2013; Bower et al., 2018). Although the cause of social isolation in this population is likely to be complex and multifaceted, evidence suggests that the amygdala is an important brain structure supporting social life in humans, and may play a role in maintaining social connection (Bickart et al., 2011, 2014).

Early experiments implicating the amygdala in social functions noted peculiar behavioral manifestations associated with bilateral removal of the anterior temporal lobes in primates (Klüver and Bucy, 1939), many of which were later credited to bilateral amygdala ablation. The amygdala is an important hub of the limbic system, and plays a key role in the processing of emotions. It has traditionally been described as being involved in fear-related processes, but more recent evidence has pointed to the amygdala’s broader contributions to a host of other complex neurobiological processes, including susceptibility to addiction (Luo et al., 2013), social judgment (Adolphs et al., 1998), social interaction (Adolphs, 2010), and decision-making (Jenison, 2014). Animal and human studies have identified the amygdala as an important structure for mediating social behavior, and part of a collection of brain networks that support social life (Bickart et al., 2014). It has been posited that subjective perception of social support is related to amygdala volume and structure (Sato et al., 2016) and that larger amygdala volume is related to larger and more complex social networks in humans (Bickart et al., 2011).

**Figure 1:**
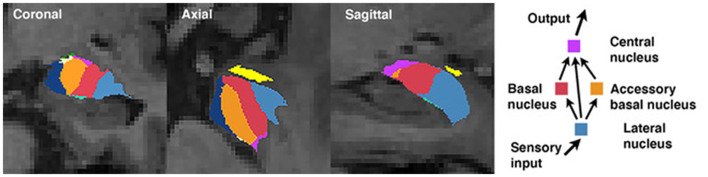
Amygdala segmentation boundaries and a simplified schematic of information processing among select nuclei.

The amygdala is comprised of a collection of nuclei that are distinguishable based on morphology, histochemistry, cytoarchitecture, and connectivity (Sah et al., 2003; LeDoux, 2007; Bach et al., 2011). A simplified model of information flow suggests the basolateral nucleus complex (consisting of the lateral, basal, and accessory basal nuclei) serves as the principal input region of the amygdala (LeDoux, 2007). The lateral nucleus is the major input nucleus for sensory information entering the amygdala, with the basal and accessory basal nuclei also acting as parallel input pathways (Manassero et al., 2018). Output connections from the basal nucleus are involved in controlling behavioral responses to stressful situations *via* connections with the striatum, and together (as part of the basolateral complex) have shown to be involved in social behaviors (LeDoux, 2007; Wellman et al., 2016). The central nucleus serves as the main output nucleus of the amygdala complex (LeDoux, 2007; Janak and Tye, 2015), receiving both direct and indirect (*via* the basal and accessory basal nuclei) projections from the lateral nucleus (Figure 1; Anglada-Figueroa and Quirk, 2005). The central nucleus has also been implicated in a host of anxiety-related and social behaviors in monkeys (Wellman et al., 2016), and output connections of the central nucleus to the brainstem are involved in controlling emotional reactions (LeDoux, 2007). Studies in primates have identified the central nucleus and basolateral nucleus complex as being important regions involved in regulating social behaviors and social network dynamics (Wellman et al., 2016), though to our knowledge no studies have explored these associations in humans.

The primary aim of this study was to examine the associations between select amygdala nuclei volumes and social network size in a group of homeless and precariously housed persons. These individuals live in a challenging environment where social networks play a critical role in accessing scarce resources. Persons in this environment are known to have small, fragmented networks (Hawkins and Abrams, 2007; Bower et al., 2018; Knerich et al., 2019), leading to social isolation and a variety of health complications (Seeman, 1996). Further, marginalized persons are exposed to a variety of risk factors (e.g., substance use, trauma) that are known to compromise the structural and functional integrity of the amygdala (Breiter et al., 1997; Morey et al., 2012). Recently, our group reported that patterns of substance use in precariously housed persons are related to the structure of their social network (Knerich et al., 2019). Such risk exposures may degrade amygdala-related functions that maintain a supportive social network, thus warranting further exploration of the amygdala as a potential neurobiological substrate for degraded social networks in a vulnerable population.

We hypothesized that smaller central nucleus and basolateral nucleus complex volumes would be associated with smaller social network size. These nuclei reflect the main input and output pathways of the amygdala and have been associated with a host of social behaviors (LeDoux, 2007; Fudge and Tucker, 2009; Wellman et al., 2016). Similar to prior work, the hippocampus was included as a control brain region (Bickart et al., 2011). In contrast to the amygdala, the hippocampal function is strongly linked with learning and memory, emotion, and spatial navigation and is not directly implicated in social behaviors and network formation. To our knowledge, this is the first study to investigate the relationship between amygdala nuclei volumes and social network size in humans.

## Materials and Methods

### Participants

The Hotel Study is an ongoing 10-year longitudinal investigation of multimorbidity in homeless and precariously housed people living in an impoverished downtown neighborhood of Vancouver, Canada (Vila-Rodriguez et al., 2013; Honer et al., 2017). Between November of 2008 and September 2010, of a potential 341 tenants, 246 (72%) individuals were recruited from four single room occupancy (SRO) hotels located in the neighborhood (Honer et al., 2017). During the interval used to establish the social network (November 1, 2009, to September 30, 2010), 201 of 246 (82%) participants provided social network data. Sixty participants had at least one social network connection and valid MRI data. All participants were 18 years or older, fluent in English, and provided written informed consent, which included consent to communicate clinically significant findings to participants’ physicians, and received honoraria. Approvals were obtained from the Clinical Research Ethics Boards of the University of British Columbia and Simon Fraser University.

### Social Network

Social support relationships were assessed using the Arizona Social Support Interview Schedule (ASSIS; Barrera, 1980). The ASSIS is a structured interview designed to assess a range of social support categories, including: (1) intimate interaction; (2) material aid; (3) physical assistance; (4) guidance; (5) social participation; and (6) positive feedback, and was previously used to study social network topology in this sample (Knerich et al., 2019). In brief, participants (ego) were prompted to report the names and identifying information of all individuals that provided social support in the past month. Supporters (alters) were identified as Hotel Study participants themselves by name, demographic information, longitudinal relational data, and verification by study staff with significant experience in the community. This yields a measure of total network size, defined as the number of people providing at least one supportive function.

A sociogram was constructed to represent the social support relationships between participants enrolled in the Hotel Study. Nodes represent individuals and the edges represent social support relationships between two individuals. For the primary analysis, participants with at least one supportive connection (edge) in the network were included. For the supplementary analysis, we examined the (egocentric) personal support networks of each participant by including all social support relationships listed by the participants.

### Clinical Measures

Demographic variables (age, sex, education, ethnicity) were self-reported during a structured baseline interview. To measure psychiatric symptoms, the Positive and Negative Syndrome Scale (PANSS; Kay et al., 1987) was administered by trained research assistants and psychiatrists. Psychiatric diagnoses were determined *via* consensus using the Best Estimate Clinical Evaluation and Diagnosis (Endicott, 1988), the Mini-International Neuropsychiatric Interview (Sheehan et al., 1998), and a mental status examination, following criteria in the *Diagnostic and Statistical Manual of Mental Disorders* (4th ed., text rev.; American Psychiatric Association, 2000). Blood samples were drawn and submitted for serological testing for HIV and hepatitis C. Everyday functioning was indexed with the Role Functioning Scale (RFS; Goodman et al., 1993) and the Social and Occupational Functioning Assessment Scale (SOFAS; Morosini et al., 2000). Self-reported history of traumatic brain injury (TBI) was recorded by asking participants if they had ever sustained a serious head or face injury. TBI was then defined as participants who responded “yes” and endorsed post-injury symptoms of either loss of consciousness, confusion, and/or memory loss.

### Neuroimaging Processing and Acquisition

Whole-brain T1-weighted anatomic images were obtained using a Philips 3T Achieva scanner equipped with an eight-channel SENSE-Head coil and using a 3D FFE T1-weighted structural sequence applied in the sagittal plane with 190 1-mm thick slices (TR/TE = 7.6/3.5 ms; acquisition matrix = 256 × 250; the field of view = 256 mm; flip angle = 8°; total acquisition time = 7:23 min). Images were visually inspected for significant motion artifact by trained raters (DJL, WS).

Amygdala nuclei were measured by quantitative morphometric analysis of T1-weighted MRI data using an automated segmentation protocol from FreeSurfer v6.0^1^ with a procedure that uses Bayesian inference with a probabilistic atlas derived from manual delineations of the amygdala using ultra-high-resolution *ex vivo* MRI scans (Saygin et al., 2017). Bilateral whole amygdala and hippocampal volumes were measured using an automated protocol from FreeSurfer v6.0. The automated segmentation of amygdala nuclei has been validated for standard resolution T1 data of varying MR contrast. The approach takes individual underlying anatomy into account, thus providing greater spatial sensitivity (Saygin et al., 2017). Segmentations were visually inspected for failures and manually corrected where necessary. All neuroimaging was completed within 1 year of social network data collection.

### Statistical Analysis

A flow chart is detailed in Supplementary Figure S1 to show how the sample was derived for the final statistical analyses, which were carried out with SPSS software (version 21.0; SPSS, Inc., Chicago, IL, USA). Social network construction and analytics were performed using the igraph package for R, version 3.5.0 (R Core Team, 2018). Network characteristics included degree centrality and assortativity. Degree centrality refers to the number of connections a node (an individual within the network) has to other nodes, whereas network homophily (also known as the assortativity coefficient) refers to the phenomenon whereby individuals are more likely to associate with others who share similar attributes. Assortativity is calculated as the fraction of connections between dissimilar and similar nodes. Sociodemographic factors (SRO residence, age, sex), total amygdala volume, and select nuclei were examined in assortativity calculations.

Multiple linear regression analyses were conducted to investigate the relationship between amygdala nuclei volumes and degree centrality using separate models for each region of interest to avoid multicollinearity. Independent variables included: amygdala nuclei volumes (central and basolateral nucleus), whole amygdala and hippocampal volumes, which were divided by total brain volume for each participant to adjust for head size. The lateral, basal, and accessory basal nuclei were summed to represent the basolateral amygdala complex. The association of each nucleus of the basolateral complex to social connection was subsequently examined in supplementary analyses. For individuals with at least one connection, social network degree centrality was entered as the dependent variable.

To capture differences between participants connected to a larger network in our sample vs. those who are more isolated, the amygdala and hippocampal volumes were compared between participants with greater than one connection and participants with only one connection using independent sample *t*-tests. In a supplementary analysis, we repeated the multiple linear regressions as outlined above using degree measured as all connections listed for each of the 60 participants, regardless of status as participants in the Hotel Study (i.e., connections not included in social network analysis). Age and sex were included as covariates. Given specific *a priori* hypotheses, no corrections were applied for multiple comparisons. Follow-up hierarchical linear regression analysis was conducted to investigate the unique effects of each nucleus on the social network connection.

## Results

The final sample included 60 participants who completed the ASSIS social network questionnaire and imaging (Supplementary Figure S1). No differences were found in demographic variables between participants with greater than one connection and participants with only one connection (Table 1). Among the clinical variables, a higher rate of methamphetamine dependence among individuals with greater than one connection was the only observed difference (χ^2^ = 5.158, *p* = 0.023). Using paired samples *t*-tests, total network size was shown to be stable across the first 18 months of the larger ongoing Hotel Study (baseline to 6 months: *t* = 0.06, *p* = 0.953; 6 months to 12 months: *t* = −0.54, *p* = 0.590; 12 months to 18 months: *t* = 0.174, *p* = 0.863).

**Table 1:** Participant demographic and clinical characteristics.

	Overall sample (*n* = 60)		1 Connection (*n* = 14)		>1 Connection (*n* = 46)		Group difference* t/*χ*^2^ (*p*)
Characteristic	*n* (%)	Mean (SD)	*n* (%)	Mean (SD)	*n* (%)	Mean (SD)	
**Age**		43.59 (10.32)		43.61 (12.18)		43.58 (9.84)	0.008 *(0.993)*
**Education**		10.40 (2.47)		10.14 (2.82)		10.48 (2.38)	−0.442 *(0.660)*
**Sex**, male	44 (73.3)		11 (78.6)		33 (71.7)		0.487 *(0.485)*
**Age of first homelessness^a^**		28.96 (11.03)		27.45 (9.95)		29.19 (11.59)	−0.560 *(0.579)*
**Duration living in neighborhood^b^**		10.02 (7.02)		9.11 (6.59)		10.30 (7.20)	−0.550 *(0.585)*
**Number of connections**		2.57 (1.59)				3.04 (1.52)	
**PANSS^c^**							
Positive		14.64 (5.83)		13.00 (3.79)		15.12 (6.27)	−1.446 *(0.159)*
Negative		16.11 (5.60)		15.92 (4.50)		16.17 (5.93)	−0.137 *(0.892)*
General		33.85 (8.00)		34.42 (7.77)		33.68 (8.15)	0.277 *(0.783)*
Total		64.60 (16.67)		63.33 (10.69)		64.98 (18.14)	−0.298 *(0.767)*
**Everyday functioning**							
RFS		12.23 (3.89)		12.43 (3.06)		12.17 (4.13)	0.213 *(0.832)*
SOFAS^d^		41.19 (13.29)		42.86 (12.60)		40.66 (13.62)	0.536 *(0.594)*
**Ever homeless^a^ Ethnicity**	29 (48.3)		7 (87.50)		22 (66.67)		2.236 *(0.135)*
						6.00 *(0.199)*
White	35 (58.3)		7 (50.0)		28 (60.9)		
Indigenous	17 (28.3)		3 (21.4)		14 (30.4)		
Other/Unknown	8 (13.4)		4 (28.6)		4 (8.7)		
**Viral infection**							
HIV	12 (20.0)		3 (21.4)		9 (20.0)		0.150 *(0.881)*
HCV^b^	42 (70.0)		9 (64.3)		33 (73.3)		−0.644 *(0.522)*
**Substance dependence**							
Alcohol	11 (18.3)		4 (28.6)		7 (15.2)		0.741 *(0.389)*
Cannabis	22 (36.7)		4 (28.6)		18 (39.1)		0.086 *(0.770)*
Cocaine	45 (75.0)		10 (71.4)		35 (76.1)		0.071 *(0.897)*
Methamphetamine	19 (31.7)		1 (7.1)		18 (39.1)		5.158 *(0.023)*
Heroin	22 (36.7)		3 (21.4)		19 (41.3)		0.823 *(0.364)*
**Self-reported history of TBI**	28 (46.7)		7 (50)		21 (45.7)		0.134 *(0.714)*

Note. N = 60; ^a^N = 41; ^b^N = 59; ^c^N = 53; ^d^N = 58. RFS, Role Functioning Scale; SOFAS, Social and Occupational Functioning Scale; HCV, hepatitis C virus; TBI, traumatic brain injury. *Comparison of groups with 1 or >1 connection.

**Figure 2:**
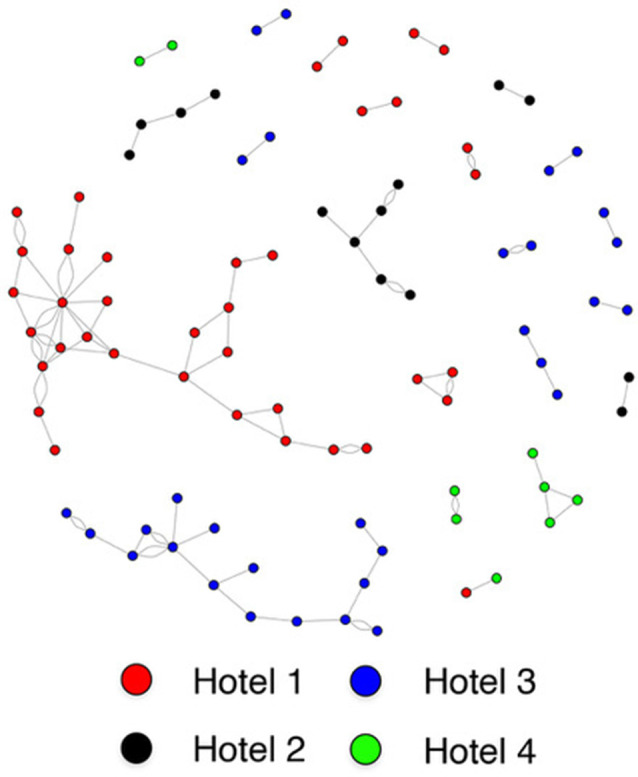
Sociogram of single room occupancy (SRO) hotel tenants.

Individuals who reside in the same SRO hotel are much more likely to form a social connection than those residing in different hotels (housing assortativity coefficient = 0.984; Figure 2). Ethnicity (assortativity coefficient = 0.212) and gender (assortativity coefficient = 0.016) homophily was not observed, nor was it observed for total amygdala (network homophily = 0.321), or central (network homophily = 0.076), basal (network homophily = 0.316), accessory basal (network homophily = 0.258) or lateral (network homophily = 0.282) nuclei volumes.

Assumptions of multivariate normality, multicollinearity, and homoscedasticity were deemed met. One univariate outlier on social network connections was identified and adjusted by assigning that case a value of one unit larger than the next most extreme value, as suggested by Tabachnick and Fidell (2013). Adjusted values are reported here. After accounting for total brain volume, individuals with larger total amygdala (*β* = 0.315, *p* = 0.031) and central nucleus volumes (*β* = 0.330, *p* = 0.010) had significantly larger network size (Table 2). Significant associations were not observed for the basolateral amygdala complex (*β* = 0.237, *p* = 0.090), or hippocampus (*β* = 0.205, *p* = 0.126). Further analysis revealed that basal nucleus (*β* = 0.331, *p* = 0.021) and accessory basal nucleus (*β* = 0.319, *p* = 0.021) volumes were significantly associated with larger network size, but not the lateral nucleus (*β* = 0.076, *p* = 0.573; Supplementary Table S1). Interactions between brain volumes and covariates (age and gender) were not significant and were not included in any of the final models.

**Table 2:** Regression results for social network connections.

	Degree (number of supportive social connections)			
Independent variable	β	R^2^	ΔR^2^	*p*-value
Block 1- *All models*		0.049	−	0.236
Age	−0.168			0.197
Sex	−0.149			0.254
Block 2- *Model 1*		0.066	0.016	0.324
Age	−0.140			0.283
Sex	−0.193			0.145
Total hippocampal volume	0.205			0.126
Block 2- *Model 2*		0.086	0.037	0.140
Age	−0.017			0.905
Sex	−0.148			0.240
Total amygdala volume	0.315			0.031
Block 2- *Model 3*		0.156	0.106	0.010
Age	−0.123			0.324
Sex	−0.129			0.297
Central nucleus volume	0.330			0.010
Block 2- *Model 4*		0.097	0.048	0.090
Age	−0.086			0.531
Sex	−0.181			0.164
Basolateral nucleus complex volume	0.237			0.090

*Note. N = 60*.

Secondary analysis using the total number of personal support network members as the dependent variable showed no significant association between central (*β* = −0.160, *p* = 0.231), basal (*β* = −0.134, *p* = 0.366), accessory basal (*β* = −0.257, *p* = 0.070), or lateral (*β* = −0.123, *p* = 0.373) nuclei volumes and network size. Whole amygdala (*β* = −0.199, *p* = 0.186) and hippocampal (*β* = −0.040, *p* = 0.774) volumes similarly showed no significant association.

## Discussion

The present study examined the associations between amygdala nuclei volumes and social network size in homeless and precariously housed persons. Consistent with previous work by Bickart et al. (2011), we found that larger total amygdala and central nucleus volumes were associated with larger social network size. Within the basolateral nucleus complex, larger basal and accessory basal nuclei were also associated with social network size, whereas the lateral nucleus volume was not. These findings support previous research identifying the amygdala as an important structure for social connection in humans (Adolphs et al., 1998; Bickart et al., 2011, 2014), and suggest certain nuclei may be differentially related to social connection in marginalized persons.

It is increasingly appreciated that the biological relevance of the amygdala can be best understood through an examination of the distinct cytoarchitectural, chemoarchitectural, and functional connectivity patterns that characterize the multiple nuclei comprising this structure (Kedo et al., 2018). A recent report showed evidence for three main subdivisions of the amygdala based on functional MRI activation patterns and their differential correlation with cortical functional networks across the brain (Sylvester et al., 2020). These functional subdivisions roughly overlap with the centromedial, laterobasal, and superficial structural partitions based on cytoarchitectonics defined by Amunts et al. (2005) and approximate the select regions we examined in the current study. Our work, therefore, represents an important extension of the correlates of amygdala nuclei from the functional neural level to the human behavioral level in a real-world social context. The central and basolateral nuclei have previously been implicated in the maintenance of social networks in primates (Wellman et al., 2016), and we showed similar associations in humans. The differential associations with social network size among the nuclei of the basolateral complex are consistent with findings from Manassero et al. (2018) showing that the basal and lateral nuclei account for opposite behavioral responses to threatening stimuli, and thus may have unique roles in their contribution to social behaviors.

Consistent with previous studies involving homeless and precariously housed individuals (Blankertz and Cnaan, 1994; Crawley et al., 2013; Bower et al., 2018), social networks in our participants were small, with approximately 60% of our sample reporting two or less supportive connections (Knerich et al., 2019). Given the well-established role of the amygdala in fear circuitry and avoidance behaviors, it is reasonable to posit that size of the social network may be related to social anxiety. Smaller volumes in the lateral and basal nuclei have been previously reported in patients with panic disorder (Asami et al., 2018). Given the high prevalence of anxiety disorders (~31–42% of individuals) observed in this sample (Vila-Rodriguez et al., 2013; Barbic et al., 2018), this may be a contributing factor to social isolation in an already challenging living environment.

Like our previous report, the social network structure in this sample is highly linked to the building of residence (Knerich et al., 2019). We further strengthened this study using egocentric network analyses that included family or distant connections outside of our study recruitment. Individuals reported few supportive connections, with almost 50% reporting four or less, which is substantially smaller than the average social network size in non-marginalized, healthy adults (Hill and Dunbar, 2003; Dunbar, 1993; Von Der Heide et al., 2014). Notably, when we expanded networks to include all individuals listed by each participant, we failed to find a significant association between amygdala nuclei and the number of social connections, raising the possibility that the neurobiological contributions to social connection may be more salient within an individual’s immediate environment. This has important clinical relevance as local social networks may be utilized to bolster treatment outcomes in this vulnerable, underserved population (Christakis, 2004). For instance, supports built directly into the immediate environment of an individual, such as on-site peer support to facilitate connections and access to health services, may be maximally effective, but this requires further investigation.

This study has several limitations. First, networks are universally dynamic and can change in unpredictable ways (Valente, 2012). This may be particularly true in homeless and precariously housed persons who typically have fragmented and fragile social networks (Green et al., 2013; Bower et al., 2018). While we showed evidence of network stability within 18 months, there may still be longer-term fluctuations in network composition. Second, variation in the local social and economic landscapes may limit the generalizability of our findings to other settings, although our sample’s characteristics are consistent with international reports on homelessness (Fazel et al., 2014). Further, comparison of the larger Hotel Study sample to other Canadian cohorts of homeless and precariously housed persons reveals substantial similarities in demographic and clinical characteristics that imply the likelihood of at least a modest generalizability (see Gicas et al., 2020). Finally, we included the hippocampus as a control brain region, similar to previous work in this area (Bickart et al., 2011), because it is not directly implicated in mediating social behaviors. However, it is important to acknowledge that there is a high rate of adverse childhood experiences among homeless persons (Liu et al., 2020). Such histories of complex trauma are likely to impact the hippocampus (Ahmed-Leitao et al., 2016); therefore, the social underpinnings of this region should not be overlooked.

This work provides a foundation for understanding the neurobiological contributions to social connection in a vulnerable and medically complex group and remains an important avenue of research amidst ongoing efforts to remove barriers to better health and wellness for persons who are homeless or precariously housed. Future research should consider the emerging models of functional amygdala-cortical interactions as a framework for understanding real-world social behaviors and how these may breakdown in the context of psychiatric illness and addiction (Sylvester et al., 2020). Building on this, the relationship between social interconnectedness and functional connectivity within the amygdala should be considered important next steps.

## Data Availability Statement

The data used in this study cannot be made publicly available because individual participant data includes identifiers which are necessary for these analyses. Further, this data cannot be publicly shared due to potential privacy infringement and related ethical and legal obligations to participants as restricted by the research ethics boards of the University of British Columbia and Simon Fraser University. Any requests should be directed to the corresponding author, Kristina M. Gicas (kgicas@yorku.ca).

## Ethics Statement

The studies involving human participants were reviewed and approved by Clinical Research Ethics Board, University of British Columbia. The patients/participants provided their written informed consent to participate in this study.

## Author Contributions

PJ and KG were involved in study conceptualization, data analysis and interpretation, and drafting of the manuscript. AT was involved in the drafting of the manuscript. All other authors were involved in data collection and/or interpretation and critically revised the manuscript for intellectual content.

## Conflict of Interest

WP reports personal fees from Abbatis Bioceuticals, MediPure Pharmaceuticals, and is owner of Translational Life Sciences. WH reports personal fees from the Canadian Agency for Drugs and Technology in Health, AlphaSights, Guidepoint, Translational Life Sciences, Otsuka, Lundbeck, and Newron, and has been a consultant (non-paid) for *In Silico*.

The remaining authors declare that the research was conducted in the absence of any commercial or financial relationships that could be construed as a potential conflict of interest.

## Footnotes

^1^http://surfer.nmr.mgh.harvard.edu/
