# Supplementary material for: Amygdala Nuclei Volumes Are Selectively Associated With Social Network Size in Homeless and Precariously Housed Persons
Source: Front Behav Neurosci. 2020 Jun 16;14:97. doi: 10.3389/fnbeh.2020.00097 (PMC7309349; doi:10.3389/fnbeh.2020.00097)
Supplement: Supplementary file 1 [file Table_1.DOCX]

**Supplementary Table 1. Regression results for social network connections and the basolateral complex nuclei.**

|  | **Degree (number of positive social connections)** | | | |
| --- | --- | --- | --- | --- |
| **Independent variable** | **β** | **R^2^** | **ΔR^2^** | **p-value** |
| Block 1- *All models* |  | .049 | - | .236 |
| Age | -.168 |  |  | .197 |
| Sex | -.149 |  |  | .254 |
|  |  |  |  |  |
| Block 2- *Model 1* |  | .055 | .005 | .573 |
| Age1 | -.155 |  |  | .246 |
| Sex | -.163 |  |  | .223 |
| Lateral nucleus volume | .076 |  |  | .573 |
|  |  |  |  |  |
| Block 2- *Model 2* |  | .136 | .090 | .021 |
| Age | -.023 |  |  | .870 |
| Sex | -.178 |  |  | .160 |
| Basal nucleus volume | .331 |  |  | .021 |
|  |  |  |  |  |
| Block 2- *Model 3* |  | .136 | .087 | .021 |
| Age | -.047 |  |  | .727 |
| Gender | -.157 |  |  | .213 |
| Accessory basal nucleus volume | .319 |  |  | .021 |

*Note.* N=60
